# Human activity has increasingly affected recent carbon accumulation in Zhanjiang mangrove wetland, South China

**DOI:** 10.1016/j.isci.2024.109038

**Published:** 2024-01-26

**Authors:** Ting Liu, Kunshan Bao, Minqi Chen, Bigyan Neupane, Changjun Gao, Claudio Zaccone

**Affiliations:** 1 School of Geographical Sciences, South China Normal University, Guangzhou 510631, China; 2 Guangdong Provincial Key Laboratory of Silviculture, Protection and Utilization, Guangdong Academy of Forestry, Guangzhou 510520, China; 3 Department of Biotechnology, University of Verona, Strada Le Grazie 15, 37134 Verona, Italy

**Keywords:** Earth-surface processes, Biogeochemistry, Global change, Aquatic science;

## Abstract

Mangrove wetlands are an important component of blue carbon (C) ecosystems, although the anthropogenic impact on organic C accumulation rate (OCAR) in mangrove wetlands is not yet clear. Three sediment cores were collected from Zhanjiang Gaoqiao Mangrove Reserve in Southern China, dated by ^210^Pb and ^137^Cs, and physico-chemical parameters measured. Results show that the OCARs in mangroves and grasslands have significantly increased by 4.4 and 1.3 times, respectively, since 1950, which is consistent with the transformation of organic C sources and the increase of sedimentation rate. This increment is due to increased soil erosion and nutrient enrichment caused by land use change and the discharge of fertilizer runoff and aquaculture wastewater. This study provides clear evidence for understanding the changes in organic C accumulation processes during the Anthropocene and is conducive to promoting the realization of C peak and neutrality targets.

## Highlights

• The C/N/P accumulation rates have been increasing since 1950
• Aquaculture waste discharge and land use change increased the sedimentation rate
• Eutrophication increased the contribution of organic C from mangrove sources
• Biological factors, sediment properties and supply influenced organic C accumulation

## Graphical abstract

**Figure:**
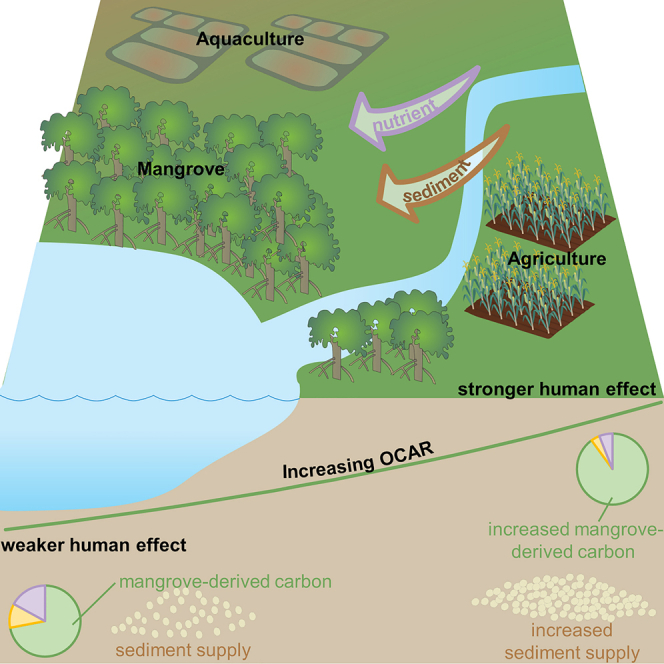

## Introduction

Mangroves have high primary productivity and well-developed roots, which can promote the sedimentation of suspended solids. Oxygen deficiency and sulfate-rich conditions slow down carbon (C) mineralization, which can be preserved in mangroves for thousands of years.^1^^,^^2^ This makes mangroves the most C-rich forests in the tropics,^3^ reducing greenhouse gas emissions and effectively mitigating the impacts of climate change.^4^

The C pool of mangroves consists of sediment C, living and dead biomass including leaves, stems, branches, litter, and roots over the short term.^5^ Sediment C is much larger than biomass C, accounting for about 49–98% of the total C stock of mangrove ecosystems.^3^ Globally, sediment organic C stock and accumulation rates in mangroves are spatially highly heterogeneous and are related to biological factors, geomorphology settings, hydrology, and physical and chemical parameters.^6^^,^^7^

Recent studies have found that human management has altered the physical and chemical properties and organic matter (OM) sources of mangrove sediments, gradually becoming a driving factor for the accumulation of organic C in mangroves.^8^^,^^9^ Over the past 50 years, mangroves have been large-scale reclaimed for agriculture and aquaculture, resulting in a loss of mangrove area.^10^^,^^11^ Moreover, when mangroves are converted to other land use types, they not only lose the ability to store C from the atmosphere but also lead to the removal of C from the sediment in the form of carbon dioxide (CO_2_).^12^ At the same time, along with the discharge of fertilizer runoff and aquaculture wastewater, nutrient overloads occurred.^13^^,^^14^ However, the impact of eutrophication on organic C accumulation is still unclear. Nutrient enrichment has been suggested in some regions to increase the deposition of phytoplankton and benthic microalgae,^15^ as well as stimulate microbial respiration and mineralization of OM,^16^ which reduced the ability of local mangrove communities to retain organic C.^17^ But for nutrient-limited mangrove ecosystems, nitrogen (N) and phosphorus (P) inputs are essential to promote sediment C inputs by stimulating plant growth.^18^ Eutrophication not only changes the dynamics of organic C by affecting plant biomass and microbial processes, but also drives the marsh loss due to the reduction of geomorphic stability.^19^ Other human disturbances, such as river damming, reservoir construction, and deforestation, can also alter the salinity and supply of sediment.^20^^,^^21^ In summary, the interactions between multiple anthropogenic activities are very complex; thus, understanding the changes in organic C accumulation rate (OCAR) and sources is fundamental to predicting the C sequestration potential in mangrove wetlands.

A large area of mangrove wetlands is distributed along the coast of Southern China. Zhanjiang City in Guangdong Province is home to China’s largest mangrove nature reserve. Over the past few decades, 5,587 ha of aquaculture ponds have been spread around its edges.^22^ At the end of each farming cycle, nutrient-rich pond sediments are discharged through the gates into the adjacent mangroves, making them a sink for nutrients and pollutants and causing serious environmental risks.^22^^,^^23^

We hypothesize that human activity, including regional aquaculture and land use change, has affected the C accumulation rate of Zhanjiang mangrove wetlands. This impact has increased with urbanization and aquaculture development over the past decades. To test such a hypothesis, we determined the accumulation rates of organic C and nutrients of three ^210^Pb-dated sediment cores, investigated their source changes by C and N isotopes and C/N ratio, and assessed the impact of anthropogenic environmental changes on the C sequestration capacity. Obtained results could help clarify the impact of human interference on the C sequestration capacity of mangrove ecosystems and provide suggestions for their protection, thereby achieving future C neutrality goals.

## Results

### Chronology and sedimentation rates

**Figure 1:**
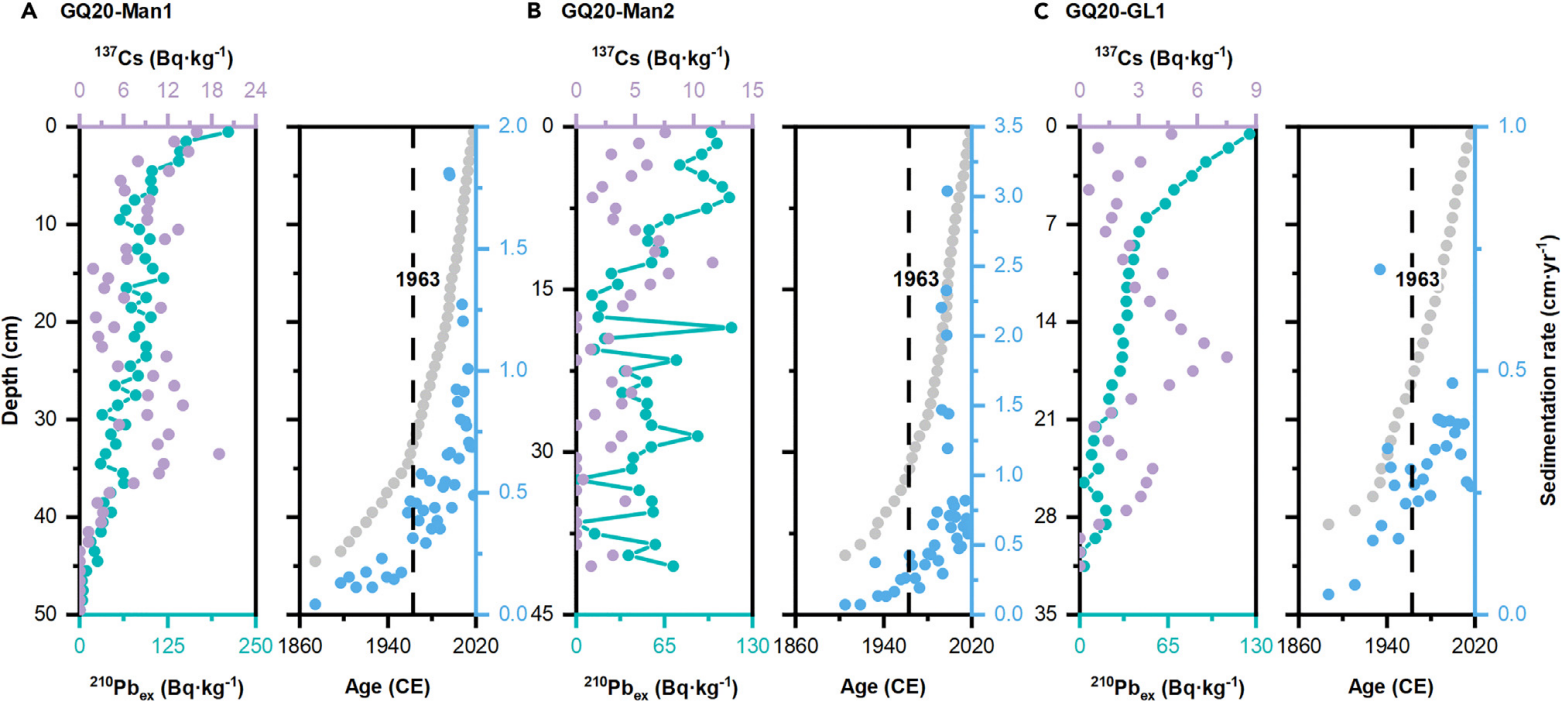
Dating results of the sediment cores from wetlands and grassland ^210^Pb_ex_ (green circles) and ^137^Cs (purple circles) activities depth profiles, age-depth model (gray circle) and sedimentation rates (blue circles) in GQ20-Man1 (A), GQ20-Man2 (B), and GQ20-GL1 (C). Chronologies were calculated using the constant rate of supply (CRS) model.

The excess ^210^Pb (^210^Pb_ex_) values in GQ20-Man1 and GQ20-GL1 varied exponentially with depth (*R*^2^ = 0.72 and 0.95, respectively) (Figure 1). The maximum ^210^Pb_ex_ values of GQ20-Man1 and GQ20-GL1 both occurred at 0.5 cm, on the surface layer, while the maximum value at GQ20-Man2 appeared at 18.5 cm. Vertical mixing occurred in the GQ20-Man2 with an irregular jagged change. The history was traced back to 1874 in GQ20-Man1, 1905 in GQ20-Man2, and 1887 in GQ20-GL1. In general, the first peak of ^137^Cs occurred in 1963, and the differences between the ages calculated in this study using ^210^Pb_ex_ were within the error range of ±10 years (Figure 1).

The sedimentation rates of GQ20-Man1 ranged from 0.04 to 1.81 cm yr^−1^, with an average of 0.57 ± 0.39 cm yr^−1^. The sedimentation rates of GQ20-Man2 were higher than that of GQ20-Man1, with a maximum value of 3.04 cm yr^−1^ and an average value of 0.72 ± 0.65 cm yr^−1^. At GQ20-GL1, the sedimentation rate was the lowest, with a mean value of 0.30 ± 0.13 cm yr^−1^

### Physical and chemical properties of the sediment

**Figure 2:**
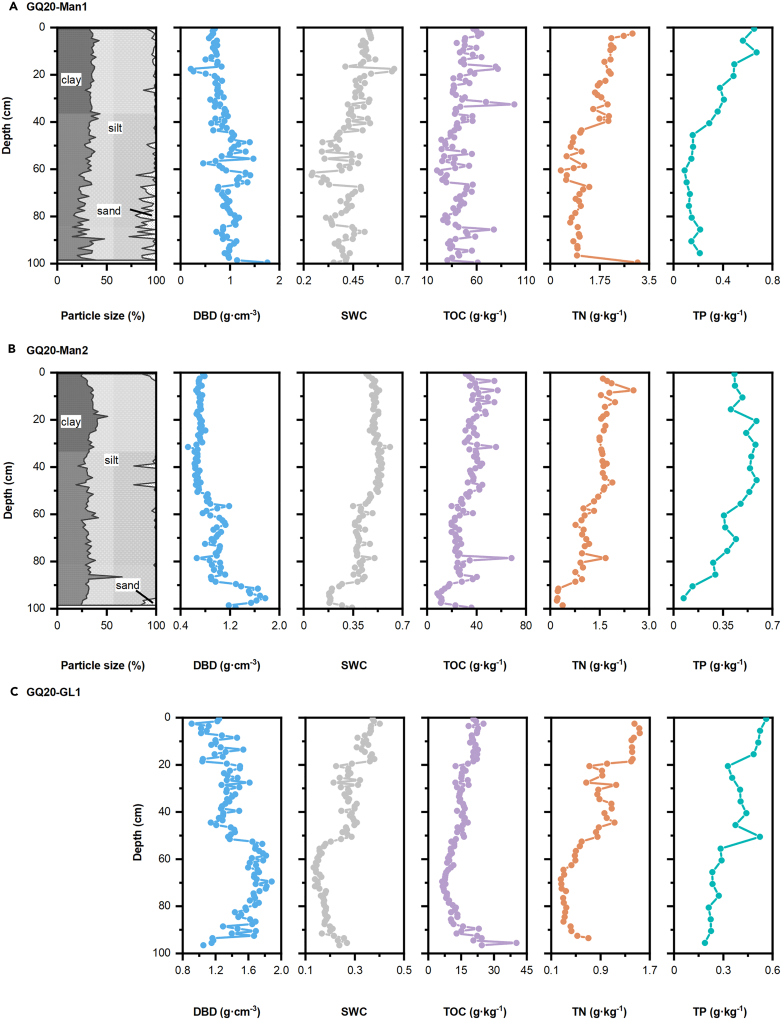
Vertical profiles of particle size, dry bulk density (DBD), sediment water content (SWC), total organic carbon (TOC), total nitrogen (TN) and total phosphorus (TP) (A) GQ20-Man1; (B) GQ20-Man2; (C) GQ20-GL1.

Except for dry bulk density (DBD), the sediment water content (SWC), and the concentration of total organic carbon (TOC) and nutrients (N and P) generally increased with depth (Figure 2). The trend of DBD, SWC, and total N (TN) of GQ20-Man1 and GQ20-Man2, collected in mangroves, were similar. Conversely, compared with GQ20-GL1 sampled in grassland areas, except for total P (TP), there were significant differences. Particle size results showed that the silt and clay content of GQ20-Man1 varied from 72.6 to 100% and increased toward the surface; GQ20-Man2 was similarly dominated by mud, and the sand content was sparse with an average value of only 1.3 ± 4.2%. The DBD ranged from 0.2 to 1.7 g cm^−3^ (avg ± S.D.: 0.9 ± 0.3 g cm^−3^) and from 0.5 to 1.8 g cm^−3^ (avg ± S.D.: 0.9 ± 0.3 g cm^−3^) in GQ20-Man1 and GQ20-Man2, respectively. The DBD of GQ20-GL1 was greater than that in the mangrove area, ranging from 0.9 to 1.9 g cm^−3^ and an average value of 1.4 ± 0.2 g cm^−3^. SWC is negatively correlated to the DBD. The range of SWC was similar to GQ20-Man1 (0.2–0.7) and GQ20-Man2 (0.2–0.6) compared to GQ20-GL1 (0.1–0.4).

GQ20-Man1 had the highest TOC content (20.1–98.1 g kg^−1^), followed by GQ20-Man2 (8.3–68.2 g kg^−1^), whereas GQ20-GL1 the lowest (6.4–40.2 g kg^−1^), with mean values of 44.0 ± 13.5, 32.4 ± 10.9 and 15.0 ± 5.6 g kg^−1^, respectively. The TN concentration of GQ20-Man1 (0.4–3.1 g kg^−1^) was higher than GQ20-Man2 (0.2–2.5 g kg^−1^) and GQ20-GL1 (0.3–1.5 g kg^−1^). Unlike TOC and TN, the TP variation range showed the following order: GQ20-Man2 (0.1–0.6 g kg^−1^) > GQ20-GL1 (0.2–0.6 g kg^−1^) > GQ20-Man1 (0.1–0.7 g kg^−1^).

**Figure 3:**
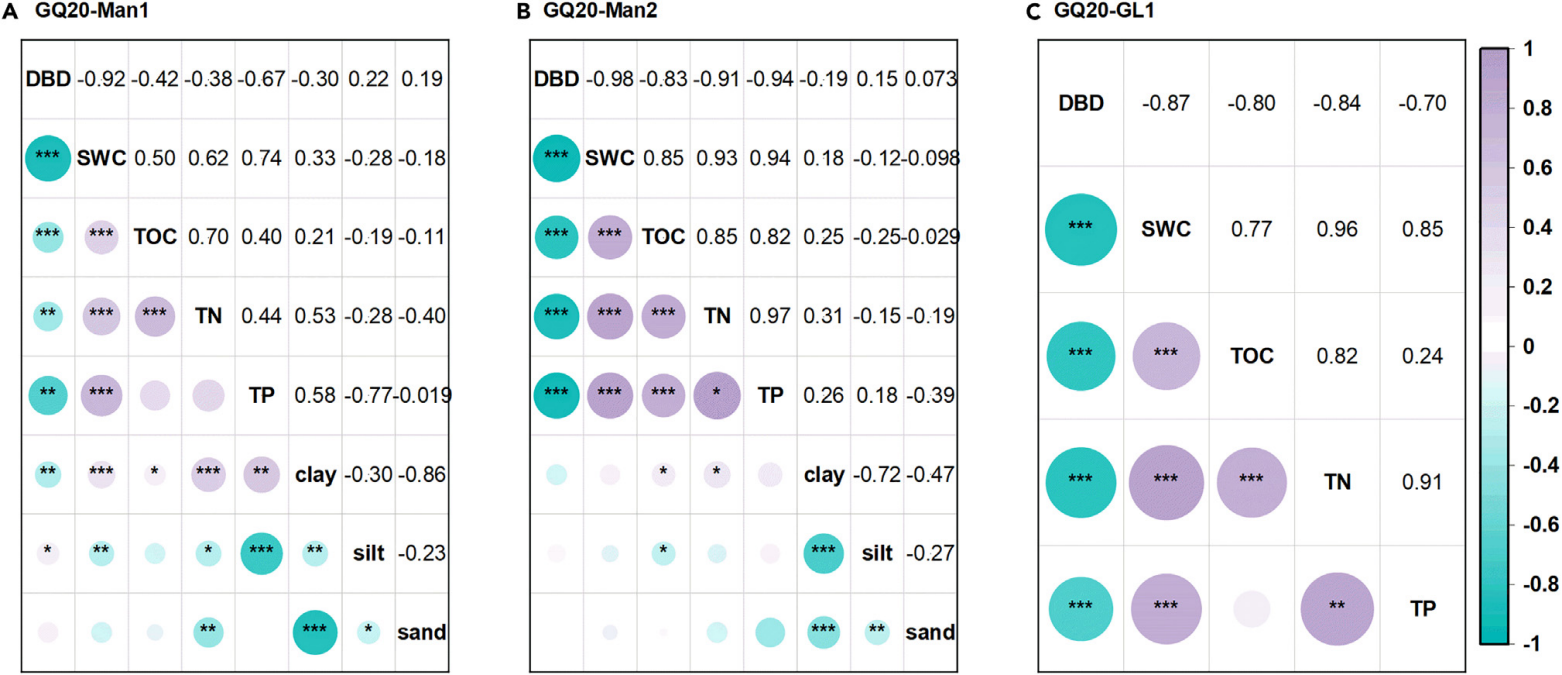
Relationships among sediment properties in mangrove and grassland (A) GQ20-Man1, (B) GQ20-Man2 and (C) GQ20-GL1. ∗, ∗∗, and ∗∗∗ represent significance of p < 0.05, p < 0.01, and p < 0.001, respectively. The size and color of the circles, as well as the number in the cells, represent the values of the correlation coefficients.

The Pearson’s correlation analysis showed that TOC content in mangrove sediment was significantly negatively correlated with DBD and positively correlated with SWC, TN and clay content (Figure 3). The correlation between TOC and TN was the strongest, followed by SWC and DBD, and the correlation with clay content was the weakest. No significant correlation was found between TOC concentration and sand content. In GQ20-Man2, TOC also showed a (significant) positive and negative correlation with TP concentration and silt content, respectively. In GQ20-GL1, the concentration of TOC showed a significantly stronger correlation with TN content, DBD and SWC.

### Stable isotopes (δ^13^C and δ^15^N) and C/N ratio

**Figure 4:**
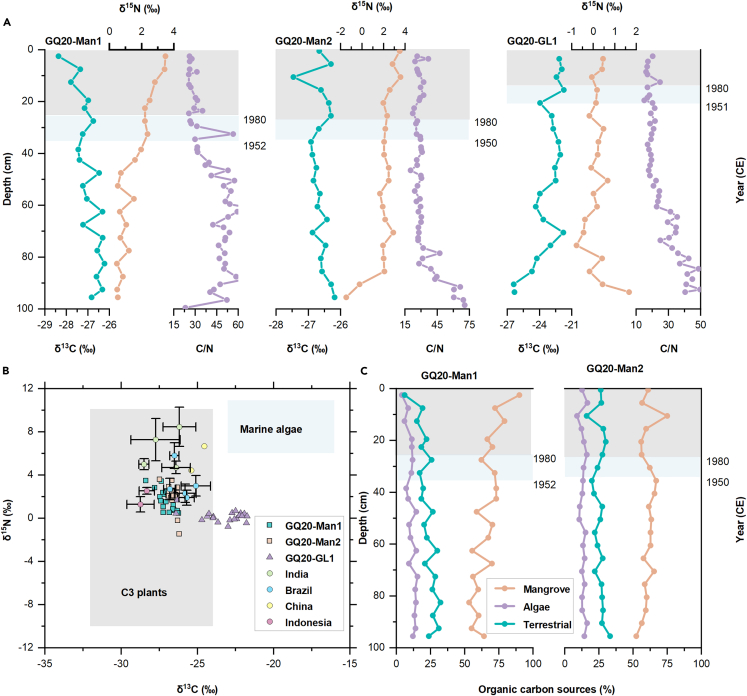
Carbon (δ^13^C) and nitrogen (δ^15^N) stable isotope signatures and C/N ratio in the sediment cores (A) Vertical profiles of δ^13^C, δ^15^N and C/N in sediment cores. (B) Comparison of δ^13^C and δ^15^N values found in the Gaoqiao area and other mangrove sites: Cochin estuary, India;^20^^,^^23^ Todos os Santos Bay and Itapessoca estuarine, Brazil;^16^^,^^17^ Qinglan Bay, China;^24^ Bintuni Bay, Indonesia.^25^ Data are reported as average ± SD. The ranges of the δ^13^C and δ^15^N values for different OM sources were established based on literature.^23^^,^^26^ C3 plants have a δ^13^C value from −32 to −24‰, a δ^15^N from −10‰ to 10‰, whereas marine algae have a δ^13^C between −16 and −23‰, a δ^15^N in the range 6‰–11‰. (C) Variations in the contributions of mangrove-derived OM, algae-derived OM, and terrestrial OM in mangrove sediment cores.

The δ^13^C and δ^15^N values of Gaoqiao mangrove sediment cores were similar to those of C3 terrestrial plants, indicating their role as main OM source (Figure 4B). The mean (±SD) δ^13^C values were −27.0 ± 0.5, −26.6 ± 0.3, and −23.3 ± 1.4‰ in GQ20-Man1, GQ20-Man2, and GQ20-GL1, respectively. For δ^15^N, GQ20-Man2 had the highest mean value (2.1 ± 1.1‰), followed by GQ20-Man1 (1.6 ± 1.0‰) and GQ20-GL1 (0.2 ± 0.5‰). The C/N ratios in GQ20-Man1 and GQ20-Man2 (41 ± 12 and 33 ± 12) were higher than those in GQ20-GL1 (26 ± 10).

Overall, downcore profiles of δ^13^C and C/N increased with depth, while δ^15^N showed a decreased tendency in mangroves. Since 1950, the δ^13^C values of mangroves have increased and have been depleted since 1980. On the contrary, the δ^15^N values have increased since 1950, especially after 1980. Both δ^13^C and δ^15^N in GQ20-GL1 exhibited oscillations and became slightly enriched after 1950. As for C/N, the values after 1950 are relatively low compared to those observed in the bottom among three sites.

The source mixing model based on δ^13^C revealed that the sedimentary OM is mainly derived from the mangrove production, followed by terrestrial sources, while marine algae contribution was the lowest (Figure 4). The contribution of mangrove production in GQ20-Man1 has increased from 53% at the bottom to 90% at the surface, with a decrease in OM from both terrestrial and marine sources, especially since 1980. The contribution of GQ20-Man2 to mangrove production showed a trend of first increasing and then decreasing over time. Taking 1950 as the breaking point, the contribution of OM from terrestrial sources has increased recently, reaching 30% in the early 1990s. OM from mangrove-derived sources decreased between 1950 and 1980, followed by a slight increase.

### Changes in C/N/P accumulation rates

The organic C stocks were 375.4 Mg ha^−1^ in GQ20-Man1, 260.9 Mg ha^−1^ in GQ20-Man2, and 206.5 Mg ha^−1^ in GQ20-GL1. The mean OCAR, TN accumulation rate (TNAR), and TP accumulation rate (TPAR) values of GQ20-Man1 and GQ20-Man2, both located in the mangrove area, were 199.4 ± 154.7, 7.9 ± 5.4, and 2.6 ± 2.6 g cm^−2^·yr^−1^, respectively. Relatively lower values (i.e., 75.9 ± 27.8, 4.6 ± 2.0, and 2.2 ± 0.9 g cm^−2^·yr^−1^, respectively) were found for GQ20-GL1 located in the surrounding grassland.

**Figure 5:**
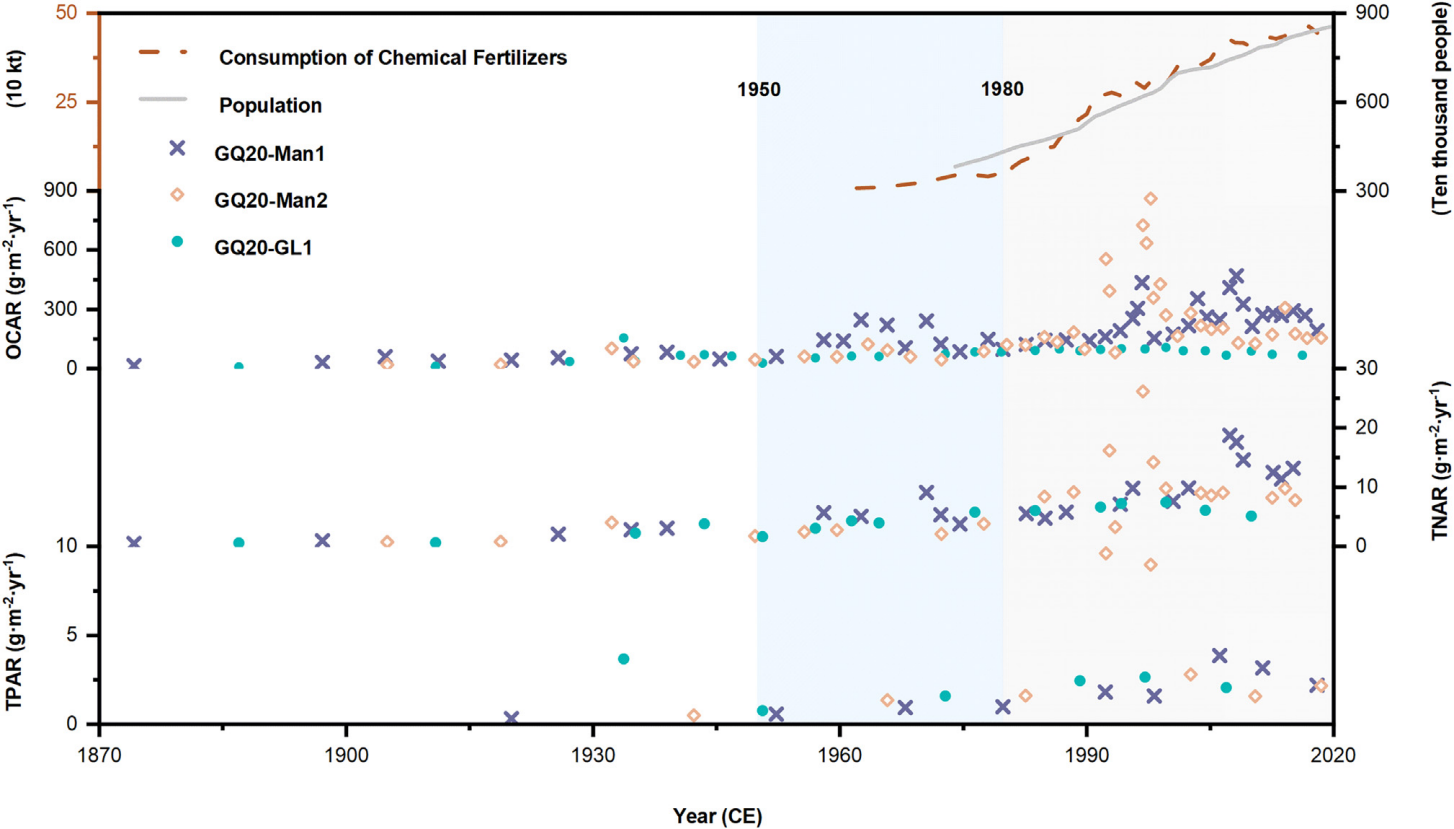
Changes in C/N/P accumulation rates (g·m^−2^·yr^−1^) of sediment cores The straight and dotted lines represent the population and consumption of chemical fertilizers changes in Lianjiang City, respectively (see also Figure S1).

C/N/P accumulation rates at three cores showed an increasing trend since 1950, particularly after 1980, which is consistent with the increase of population and fertilizer consumption of Liangjiang City (Figure 5). Kruskal-Wallis ANOVA analysis was used to determine C/N/P accumulation rates before 1950, between 1950 and 1980, and after 1980. Results indicated significant differences (p < 0.05): in fact, compared to the period 1900–1950, the C/N/P accumulation rates in the mangrove region increased by 5.3, 4.8, and 8.6 times, respectively, after 1980. In comparison, the OCAR of grassland areas increased by 1.4 times, TNAR by 2.9 times, while TPAR decreased (0.7 times), indicating that mangroves accumulate more organic C and nutrients than grassland ecosystems. There were significant differences in OCAR and TNAR among the three sites (p < 0.05), except TPAR since 1950.

**Figure 6:**
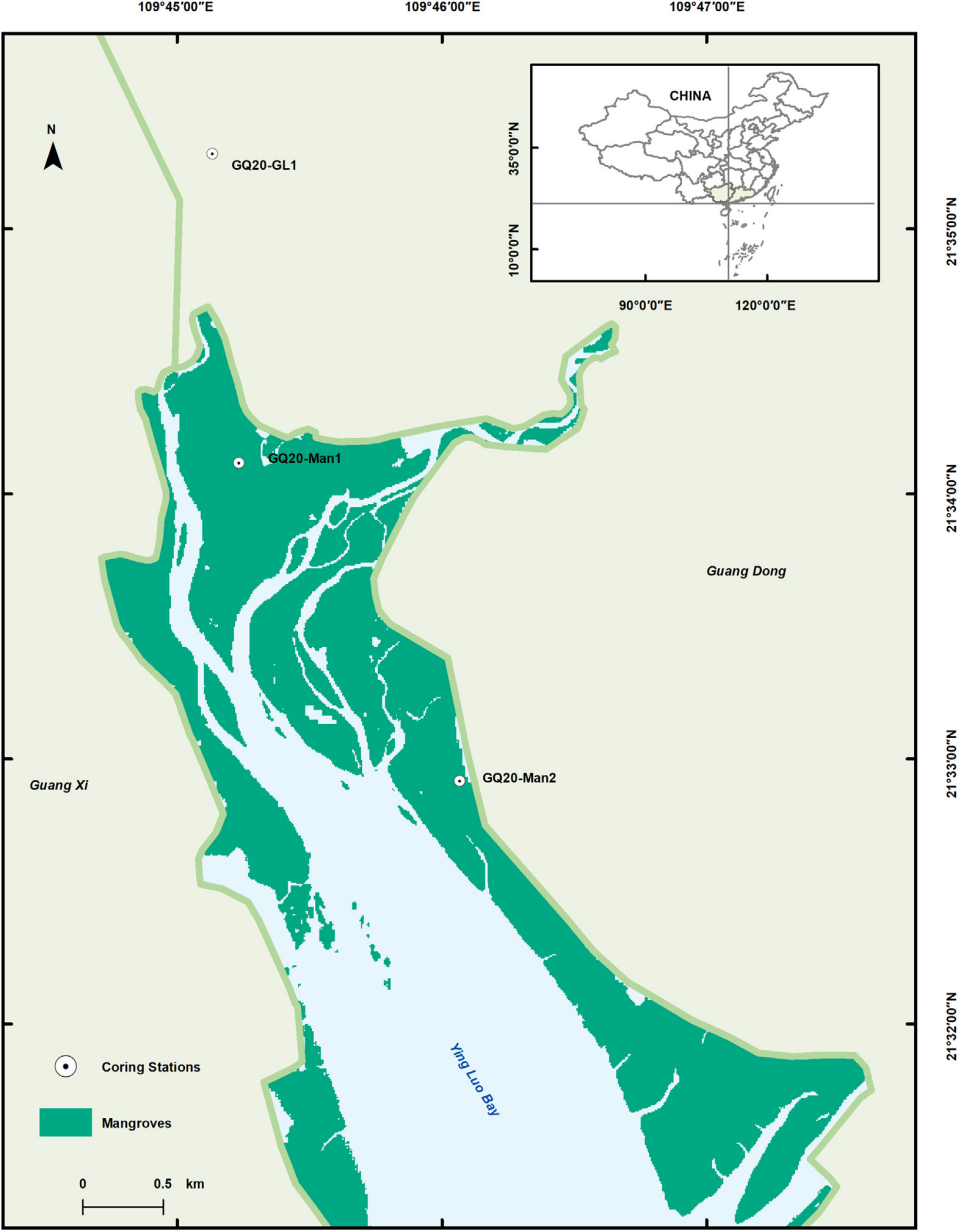
Map of the core area of Zhanjiang Mangrove Nature Reserve (ZMNR), Guangdong Province, China The dots are the sampling locations.

## Discussion

### Chronology and sedimentation rates

The values of ^210^Pb_ex_ in GQ20-Man1 and GQ20-GL1 varied exponentially with depth (Figure 2), indicating that the sediment environment was stable.^24^ Due to enhanced or dilutive effects from changes in sediment supply, and some biotic or physical disturbance, the constant rate of supply (CRS) model was used for calculation. This is more reasonable because the CRS model is calculated based on the total accumulation of ^210^Pb_ex_ and is not sensitive to the effects of vertical mixing.^25^^,^^26^

The average sedimentation rate of Gaoqiao mangrove area is higher than that at a global scale (0.28 cm yr^−1^),^27^ and also higher than the average of mangroves affected by urban effluents (0.55 ± 0.20 cm yr^−1^).^28^ Since 1950, the sedimentation rate of Gaoqiao mangrove wetland has been increasing. On the mesoscale, the sedimentation rate in this ecosystem is determined by hydrodynamics and sediment supply.^29^ Land use changes in catchment areas can alter the transport of suspended sediment and particulate OM. The reduction of forest area upstream of rivers and the expansion of agriculture may lead to increased soil erosion, thereby increasing the sedimentation rate of mangroves.^30^^,^^31^ In addition, dense aquaculture ponds are distributed outside the Gaoqiao mangrove wetland; consequently, after the seasons shrimp harvest, organic materials such as fish bait and animal waste are discharged into the mangroves area,^32^ thus contributing to the increase in sedimentation rate.^33^ Mangroves in Mexico showed an exponential increase in sediment mass accumulation rates due to the intensification of continental erosion caused by land use and population growth.^26^ In Puerto Rico, mangrove sedimentation rate at Caño Martin Peña, the most urbanized area, has varied significantly in recent decades compared to historical decades.^34^

### Site differences of organic C accumulation

The spatial variability of sediment organic C stocks and accumulation rates in mangroves is very large.^2^^,^^27^ TOC accumulation in mangroves is a balance between production, export and decomposition, which in turn is influenced by a combination of biological factors (e.g., primary production, benthic organisms, and species composition), geomorphic environment (e.g., sedimentation rate, mineral and organic sediment input, tidal difference, and sea level change), and sediment physical and chemical properties (e.g., nutrient content and pH).^35^^,^^36^^,^^37^

Sediment properties are considered the main factor controlling TOC content in mangrove sediment, as well as TN content, SWC and sediment texture.^38^^,^^39^ TN in sediments is closely coupled with TOC and influences the input of OM by affecting plant growth.^39^^,^^40^ An increase in SWC, which generally mirrors a lower DBD,^41^ will inhibit the diffusion and penetration of oxygen and affect microbial metabolism.^38^ This leads to a decrease in the redox reaction potential and decomposition rate, resulting in an increase of TOC content in sediment.^42^ The physical preservation of TOC by clay particles can slow down the decomposition of microorganisms,^38^ but the effect of clay content on TOC was weaker than that of SWC and TN content in Gaoqiao mangrove wetlands. The role of other sediment properties such as pH and salinity cannot be ignored and further exploration is needed.^43^

Often, elevated primary productivity can lead to higher debris input and fine root turnover rates.^44^ GQ20-Man1 is located in the high-value area of gross primary production (GPP), while GQ20-Man2 has a lower GPP.^44^ Primary productivity is often positively correlated with species age.^45^ Mangroves of GQ20-Man1 are older than GQ20-Man2, with longer-lasting inputs of litter and roots, which could promote organic C accumulation.^46^

Unlike terrestrial ecosystems, the Gaoqiao mangrove wetland belongs to the estuarine type, with GQ20-Man1 and GQ20-Man2 distributed in the intertidal zone, receiving supply from tidal and river sediments (Figure 6). High OCARs are typically found in mature forests in river deltas and severely affected catchments^47^ due to high primary productivity, allochthonous source inputs, and sedimentation rates.^48^^,^^49^ Because of the higher sedimentation rates of GQ20-Man2, the OCAR was greater than GQ20-Man1, while the organic C stock was lower than GQ20-Man1 due to the dilution effect.^50^ A significant linear positive correlation between OCAR and mass accumulation rate (MAR) is found, whose correlation coefficient is greater than that between OCAR and TOC (Figure S1). It indicated that the sedimentation rate of the Gaoqiao mangrove is a more important factor affecting the OCAR than the TOC.^51^

### Identification of sediment organic C sources

The sources of OM in mangroves typically include local production and external inputs. The former mainly comes from the litter, roots, and benthic microalgae of mangroves, while the latter comes from the phytoplankton and suspended sediments carried by tidal and river inputs.^38^^,^^52^ In general, C3 plants (including those characterizing mangroves) have a δ^13^C value from −32 to −24‰, a δ^15^N from −10‰ to 10‰, and a C/N > 20, whereas marine algae have a δ^13^C between −16 and −23‰, a δ^15^N in the range 6‰–11‰, and a C/N ratio between 4 and 10.^49^^,^^53^^,^^54^ Therefore, the combination of δ^13^C, δ^15^N, and C/N can effectively identify the source of coastal sediments. The values we collected in Gaoqiao reflected that OM is mainly derived from C3 vegetation, consistent with previous reports.^43^

Sedimentary δ^15^N has also been used to reconstruct the impact and historical changes of human activities on N accumulation.^17^^,^^55^ For example, human sewage input, fertilizers, feces, and other allochthonous OM sources are usually enriched in the heavier ^15^N.^56^ In addition to the effects of N source input, δ^15^N signatures are often determined by complex biogeochemistry processes (including N fixation, remineralization, nitrification, volatilization, and denitrification).^57^ Therefore, the increase in δ^15^N in recent sediments may be related to increased denitrification and inputs from aquaculture wastewater and farm runoff.^28^^,^^56^^,^^58^^,^^59^ Relative to mangroves, grasslands had lower δ^15^N and stable vertical variation, reflecting the weaker influence of human activities on GQ20-GL1.

In recent sediments, C/N of GQ20-Man1 has begun to decrease, possibly due to N adsorption from human sources into the sediment.^60^ The low value of C/N may also be related to the transformation of OM sources, passive leaching and microbial decomposition during early diagenesis, resulting in a greater loss of C than N.^61^^,^^62^ Afterward, there was almost no change in C/N, indicating that C and N degradation pathways were consistent at the same rate during this period.^63^ At the bottom, the increased C/N of GQ20-Man2 and GQ20-GL1 may be attributed to the preferential loss of N-rich OM.^60^

To sum up, compared with C/N and δ^15^N, the “conservative” of δ^13^C in identifying sources is not inhibited by the decomposition of OM.^53^^,^^64^ An endmember mixing model was established using δ^13^C to quantitatively estimate the contributions of terrestrial, mangrove, and algae sources to mangrove sediments. The slight positive deviation of the δ^13^C value between 1950 and 1980 indicates an increase in the contribution of terrestrial and algae sources, possibly induced by deforestation and agricultural expansion.^65^^,^^66^ After 1980, increased nutrients indicated by enriched δ^15^N promoted litter and root deposition of mangroves,^40^^,^^67^^,^^68^ resulting in lower δ^13^C values. On the other hand, the protection and restoration of mangroves, and the flourishing and expansion of mangroves, are also the reasons for the increase in the OM from mangrove sources.^69^^,^^70^^,^^71^

### Human impacts on trends of C accumulation rates

The TPAR of Gaoqiao mangrove is higher than the global average of 0.5 g m^−2^·yr^−1^, while the TNAR is lower than the global average of 8.9 g m^−2^·yr^−1^.^72^ However, in recent decades, the TNAR of GQ20-Man1 reached 18.8 g m^−2^·yr^−1^. High nutrient fluxes are related to the discharge of farmland fertilizers and aquaculture wastewater.^23^ After 1980, the application of chemical fertilizers in Lianjiang City increased sharply, and the government advocated for aquaculture. Therefore, reclamation aquaculture became very popular in the 1990s. The sediment discharged from shrimp ponds carries unabsorbed nutrients into the mangrove forest, making it a sink of nutrients.^73^

The OCAR in mangroves affected by human activities reached 1,023 g m^−2^·yr^−1^ in the Cubatão mangroves in Brazil,^15^ but there are also mangroves with much lower values worldwide (Table 1).^17^^,^^74^ This is because the input of nutrients simultaneously stimulates the production and decomposition of OM in the sediment, and the impact on mangroves depends on the degree of nutrient enrichment and the nature of the sediments themselves.^9^ However, some studies have shown that nutrient availability hampers C storage by increasing the availability of mineral nutrients for decomposers and altering litter composition.^75^ However, N addition can also induce changes in microbial communities and sediment acidification to inhibit the production of oxidase and microbial metabolism, thereby delaying sediment C decomposition.^76^ In addition, nutrient enrichment promotes root and branch growth and expansion in leaf area,^40^^,^^67^ increases aboveground and belowground biomass, fixing more C from the atmosphere to the soil.^77^^,^^78^ Nutrients can also stimulate the growth of algae, which can be deposited into sediment along with the original mangrove litter.^15^^,^^79^ Thus, the increased OCAR in nutrient-limited Gaoqiao mangrove sediments can be partially attributed to the contribution of nutrient inputs to organic C sequestration, as indicated by the positive correlation among TOC, TN, and TP (Figure 2). The OM inputs, mainly plant or root biomass, to sediment are mostly composed of labile, particulate fractions that are more prone to a rapid decomposition. Thus, although the development of mangroves increased TOC, it also resulted in greater amounts of labile organic C fractions in mangroves^80^; as a consequence, it is necessary to improve the research on the mechanism of sediment organic C stabilization, which is a key path to increase the blue C sinks in coastal zones. Eutrophication has also been proven to contribute to marsh loss,^19^ in addition to focusing on the dynamics of organic C accumulation, the coastal landscape conversion cannot be ignored.

**Table 1:** C/N/P accumulation rates in mangrove wetlands around the world

Region		OCAR (g·m^−2^·yr^−1^)	TNAR (g·m^−2^·yr^−1^)	TPAR (g·m^−2^·yr^−1^)	Year	Reference
China	ZMNR	193.82 ± 108.57	8.36 ± 4.70	1.72 ± 1.13	1900–2018	This study
	ZMNR	205.44 ± 192.99	7.54 ± 5.91	3.58 ± 3.36	1900–2018	This study
	Jiulongjiang Estuary	NA	29.7	18.8	NA	Along et al., 2005^81^
India	Cochin River estuarine	835	65	73	1990–2018	Passos et al., 2022^21^
	Cochin River estuarine	360	24	48	1949-1990	Passos et al., 2022^21^
Brazil	Suape estuary system	133.18 ± 65.97	5.34 ± 2.63	NA	1979–2016	Passos et al., 2021^74^
	Suape estuary system	114.83 ± 62.80	2.74 ± 1.32	NA	1860–1979	Passos et al., 2021^74^
	Vitória Bay	388	14	NA	NA	Bernardino et al., 2020^82^
	Itapessoca estuarine	119.94	4.11	3.62	NA	Passos et al., 2022^17^
	Cubatão	1023	31	17	1954–2014	Sanders et al., 2014^15^
	Sepetiba Bay	783.47 ± 103.97	61.6 ± 7.4	14.57 ± 0.47	1900–2000	Pérez et al., 2020^83^
Mexico	Everglades National Park	123 ± 19	7.0 ± 1.4	0.20 ± 0.08	NA	Breithaupt et al., 2014^72^
Peru	Zarumilla Creek	192.5 ± 18.0	11.5 ± 0.8	4.48 ± 0.7	1940–2014	Pérez et al., 2020^79^
	Gallegos Creek	403.3 ± 127.7	24.7 ± 8.3	7.52 ± 0.7	1940–2014	Pérez et al., 2020^79^
Indonesia	Berau	1722 ± 183	NA	NA	NA	Kusumaningtyas et al., 2019^84^
	Central Segara Anakan Lagoon (SAL)	658 ± 311	NA	NA	NA	Kusumaningtyas et al., 2019^84^
	Eastern SAL	194 ± 46	NA	NA	NA	Kusumaningtyas et al., 2019^84^
	Bintuni Bay	90	NA	NA	NA	Sasmito et al., 2020^35^
World	NDL	NA	8.9	0.5	NA	Breithaupt et al., 2014^72^
	IDL	NA	12.5	6.5	NA	Breithaupt et al., 2014^72^
	NA	163	NA	NA	NA	Breithaupt et al., 2012^27^
	Conserved mangrove	NA	5.8 ± 2.1	0.8 ± 0.5	NA	Pérez et al., 2021^28^
	Affected mangrove	NA	21.5 ± 8.6	17.9 ± 2.4	NA	Pérez et al., 2021^28^

ZMNR, Zhanjiang Mangrove National Nature Reserve; NA, not available; NDL, non-anthropogenically disturbed locations; IDL, included anthropogenic disturbed locations. Data are reported as average ± SD.

In conclusion, identifying the C sequestration potential of mangroves plays an important role in implementing ecological restoration, formulating management policies, and achieving the “dual C” goals. This study combined the ^210^Pb and ^137^Cs dating methods and then used δ^13^C and δ^15^N, to investigate the changes in the rate and source of organic C accumulation in the Zhanjiang mangrove wetland over the past century, as well as the underlying mechanisms.

The spatial distribution characteristics of organic C accumulation in mangrove wetlands are jointly determined by primary productivity, sedimentation rate, and sediment properties. After 1950, the OCAR initially increased and sharply increased after 1980, consistently with the variations in population and agricultural development in Zhanjiang City. At the same time, the agricultural expansion and reclamation in the adjacent areas had impacts on the source of OM. Since 1980, the input of nutrients has promoted the development of mangrove biomass, increasing the contribution of OM from mangrove sources to sediment. However, compared to organic C input, the high sedimentation rate caused by land use transformation had a more significant effect on organic C sequestration. Finally, investigating the stabilization mechanisms allowing a long-term preservation of OM in sediment is of great significance for increasing the C sequestration capacity of coastal wetlands.

### Limitations of the study

Although stable isotopes have been widely used to quantify the relative contribution of external and local organic C sources in coastal ecosystems, there is a cross overlap between the δ^13^C values of mangrove and terrestrial plants. In subsequent research, it would be useful to separate multiple inputs from mangrove sediments by combining specific taxonomic biomarkers, such as lipids and lignin, unique to mangroves. Moreover, collecting more samples and exploring the effects of other soil properties, climate, and human activities on organic C accumulation by combining random forest model, structural equation model, and redundancy analysis would contribute to better understand the role of mangrove environments to (C, N, and P) global biogeochemical cycles.

## STAR★Methods

### Key resources table

**Table:** 

REAGENT or RESOURCE	SOURCE	IDENTIFIER
**Deposited data**		

population in Lianjiang City	The people’s Government of Zhanjiang Municipal	https://www.zhanjiang.gov.cn/zjsfw/bmdh/tjxxw/zwgk/tjsjzl/tjnj/content/post_1840795.html
consumption of chemical fertilizers in Lianjiang City	The people’s Government of Zhanjiang Municipal	https://www.zhanjiang.gov.cn/zjsfw/bmdh/tjxxw/zwgk/tjsjzl/tjnj/content/post_1840795.html

**Software and algorithms**		

Isosource	Phillips et al.^85^	https://www.epa.gov/sites/default/files/201511/isosourcev1_3_1.zip

### Resource availability

#### Lead contact

Further information and requests can be directed to the lead contact, Claudio Zaccone (claudio.zaccone@univr.it).

#### Materials availability

This study did not generate new physical materials.

#### Data and code availability

• This paper analyzed publicly available data. These accession numbers for the datasets were listed in the key resources table.
• The article does not report any new code.
• Any additional information required to reanalyze the data reported in this article is available from the lead contact on request.

### Method details

#### Study area and sampling

ZMNR (20°14′ - 21°35′ N, 109°40′ E − 110°35′ E), located on the northeast coast of Beibu Gulf and the northwest of Leizhou Peninsula, was established in 1990. It is the largest coastal wetland nature reserve, characterized by many species and the highest concentration of mangroves in China. Gaoqiao mangrove is located in the core area of the ZMNR, with dense shrimp ponds distributed at the edges.^86^ The average pH value is 7.24, and the average content of TP and TN in the surface water exceeded the limit values of the surface water V category.^68^ The annual average temperature is 23.8°C, and the annual average rainfall is 1704 mm.^87^ Species such as *Aegiceras corniculatum*, *Bruguiera gymnorrhiza*, *Avicennia marina*, and *Rhizophora stylosa* grow in the study area. The region is characterized by irregular tides, with an average tidal range between 2.53 and 6.25 m.^88^ It is a typical estuarine mangrove forest with the inflow of the Gaoqiao River, Jiangbei River, Maizhao River, and Ximi River.^89^

In November 2020, two 100-cm depth sediment cores, namely GQ20-Man1 and GQ20-Man2, were collected in the high intertidal zone of the Gaoqiao mangrove wetland, and a 97-cm depth sediment core GQ20-GL1 in the surrounding grassland, using a gravity sampler (Figure 6). Subsequently, sediment cores were divided into 1-cm slices and placed in polyethylene plastic bags for storage at −18°C. The dominant plant species of GQ20-Man1 and GQ20-Man2 were *Bruguiera gymnorrhiza* and *Rhizophora stylosa,* respectively, while that of GQ20-GL1 was *Cynodon dactylon*.

#### Physical properties

Particle size analysis was carried out by Laser Diffraction using a Malvern Mastersizer 3000 (Malvern, England). Aliquots of 10% H_2_O_2_ and 10% HCl were added to the sample to remove OM and carbonates, respectively. A solution of sodium hexametaphosphate (Na_6_[(PO_3_)_6_]) was added to disperse the sample. The obtained grain size results were classified to fractions of clay (<4 μm), silt (4–63 μm) and sand (>63 μm).^89^ Samples were freeze-dried for 24 h and ground in an agate mortar after removing the roots. The DBD was determined as the ratio between the dry sample weight and the corresponding volume. The SWC was determined as the sediment weight difference before and after drying over the dry sediment weight.

#### Total organic C, TN and TP

TOC and TN concentration was measured using a multi N/C 3100 and an Elementar Vario Macro cube (Hesse, Germany), respectively. Samples were treated with 2 mol/L HCl for full reaction to remove inorganic C. The values of C/N ratios were calculated as the atomic ratio of the TOC to TN. TP was measured after a mixture-acid digestion (HNO_3_/H_2_O_2_/HClO_4_, 5/1/1, v/v) using an Atomic Emission Spectroscopy with Inductively Coupled Plasma (ICP-AES Prodigy 7, Teledune Leeman Labs, USA).

#### Stable C and N isotopes and end-member mixture models

The δ^13^C and δ^15^N values were measured using MAT 253 isotope ratio mass spectrometer (Thermo Electron, Bremen, Germany) following samples acidification. Stable isotope ratios were calculated as δ^13^C = [(δ^13^C_sample_/δ^13^C_standard_)–1] × 1000 and δ^15^N = [(δ^15^N_sample_/δ^15^N_standard_)–1] × 1000. Vienna Pee Dee Belemnite and atmospheric air (Air) were used as a reference for δ^13^C and δ^15^N, respectively. The analytical precision for both δ^13^C and δ^15^N was ±0.10‰.

End-member mixture models are used to quantitatively calculate the relative contributions of different potential end members to the sample. IsoSource can calculate n isotopes and > n+1 end members. It is based on a stable isotope mass conservation model; when the source is > n+1, all possible combinations of each source are calculated by superposition in a given increment (1% in this study). The weighted average of each group and the measured value of the mixture are compared. If it is within the tolerance, it is considered a possible solution. This study selected the average δ^13^C value of mangrove leaf in Gaoqiao as the endmember of mangrove production (−29.0‰) and fluvial sediments flowing into the Beibu Gulf as terrestrial sources (−24.3‰).^90^^,^^91^^,^^92^ The algae source used the average δ^13^C value of Gaoqiao phytoplankton and benthic microalgae (−20.3‰). The contribution percentage of each C source was calculated as follows:

(Equation 1) $$\delta^{13}C_{\text{sample}}=\left(f_{M}\times \delta^{13}C_{M}\right)+\left(f_{T}\times \delta^{13}C_{T}\right)+\left(f_{A}\times \delta^{13}C_{A}\right)$$

(Equation 2) $$f_{M}+f_{T}+f_{A}=1$$

where *M* (Mangrove), *T* (Terrestrial), and *A* (Algae) are the potential OM end-members and *f* represents the contributory percentage of each endmember.

#### Age dating and chronology

^210^Pb and ^137^Cs age dating was determined by a γ spectrum analysis system, mainly composed of high-purity germanium well detector (Ortec HPGe GWL) and Ortec 919 spectrum controller (EG&G Ortec G Company). The standard samples of ^210^Pb were compared by the University of Liverpool, and the standard samples of ^137^Cs and ^226^Ra were provided by the China Institute of Atomic Energy. The CRS model was used for the calculations, according to the following equations:

(Equation 3) $$t=1/\lambda \times \ln\left(C_{0}/C_{Z}\right)$$

(Equation 4) SR=Δz/Δt

Then, the organic C stock and OCAR were calculated by the following equations:

(Equation 5) $$OCAR\left(g\cdot m^{-2}\cdot yr^{-1}\right)=TOC\left(g\cdot kg^{-1}\right)\times DBD\left(g\cdot cm^{-3}\right)\times SR\left(cm\cdot yr^{-1}\right)\times 10$$

(Equation 6) $$Organic\;C\;stock\left(Mg\cdot ha^{-1}\right)=\sum TOC\left(g\cdot kg^{-1}\right)\times DBD\left(g\cdot cm^{-3}\right)\times H\left(cm\right)\times 100$$

where *t* is the age of deposition, *λ* is the decay constant (0.03114 a^−1^), *C*_*0*_ is the total ^210^Pb_ex_ input (Bq·cm^−2^) in the sediment cores, *C*_*z*_ is the cumulative ^210^Pb_ex_ input (Bq·cm^−2^) in the core from *Z* cm depth to the bottom layer, *SR* is the sedimentation rate in each layer, *H* is the sediment thickness. Finally, 10 and 100 are both unit conversion factors.

### Quantification and statistical analysis

The results obtained were tested for normality using the Shapiro-Wilk test. Differences in sediment physical and chemical properties between mangroves and grasslands were determined using the independent samples T-test for indicators that were approximately normally distributed; otherwise, the Mann-Whitney U Test was used. The correlation between TOC and other properties was analyzed using Person linear correlation. These analyses were conducted at a 95% confidence level.
