# Supplementary material for: Human activity has increasingly affected recent carbon accumulation in Zhanjiang mangrove wetland, South China
Source: iScience. 2024 Jan 26;27(3):109038. doi: 10.1016/j.isci.2024.109038 (PMC10867414; doi:10.1016/j.isci.2024.109038)
Supplement: Document S1. Figure S1 [file mmc1.pdf]

iScience, Volume *27*

Supplemental information

Human activity has increasingly affected recent carbon accumulation in Zhanjiang mangrove wetland, South China

Ting Liu, Kunshan Bao, Minqi Chen, Bigyan Neupane, Changjun Gao, and Claudio Zaccone

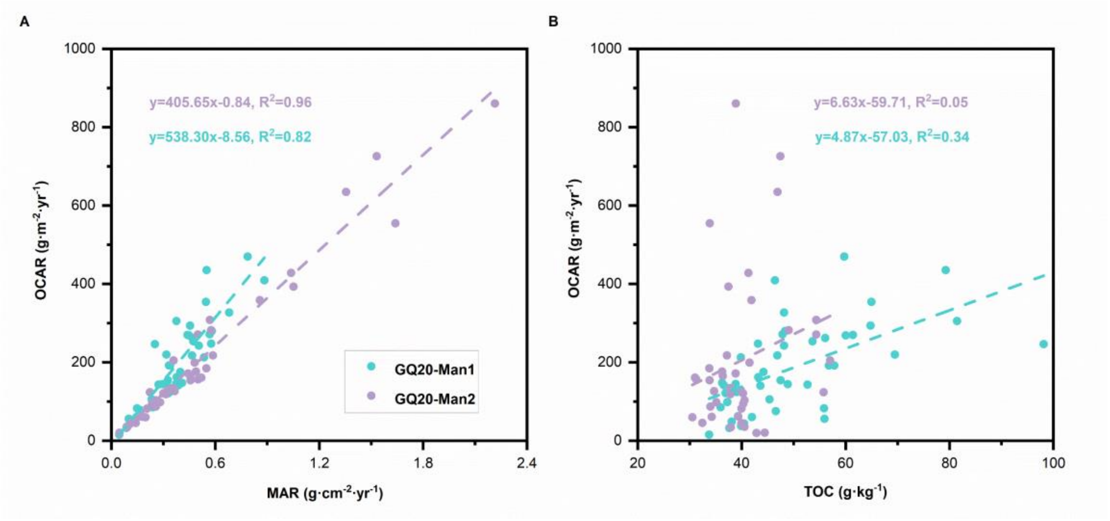

Figure S1. Correlation between OCAR and MAR/TOC in mangrove wetland, related to Figure 5. The green and purple dashed line represent the fitting curve of the regression in GQ20-Man1 and GQ20-Man2, respectively. MAR is the mass accumulation rate, obtained by multiplying SR and DBD.
